# The association between psychosocial and structural-level stressors and HIV injection drug risk behavior among Malaysian fishermen: A cross-sectional study

**DOI:** 10.1186/s12889-016-3125-7

**Published:** 2016-06-02

**Authors:** Lynn Murphy Michalopoulos, Tina Jiwatram-Negrón, Martin K. K. Choo, Adeeba Kamarulzaman, Nabila El-Bassel

**Affiliations:** Social Intervention Group, Global Health and Mental Health Unit of the Social Intervention Group, Global Health Research Center of Central Asia, Columbia University, School of Social Work, New York, NY USA; Social Intervention Group, Global Health Research Center of Central Asia, Columbia University, School of Social Work, New York, NY USA; Centre of Excellence for Research in AIDS (CERiA), Faculty of Medicine, University of Malaya, Kuala Lumpur, Malaysia

**Keywords:** Injection drug use, Fishermen, Malaysia, Depression, Policing, HIV risk

## Abstract

**Background:**

Malaysian fishermen have been identified as a key-affected HIV population with HIV rates 10 times higher than national rates. A number of studies have identified that psychosocial and structural-level stressors increase HIV injection drug risk behaviors. The purpose of this paper is to examine psychosocial and structural-level stressors of injection drug use and HIV injection drug risk behaviors among Malaysian fishermen.

**Methods:**

The study employs a cross-sectional design using respondent driven sampling methods. The sample includes 406 fishermen from Pahang state, Malaysia. Using multivariate logistic regressions, we examined the relationship between individual (depression), social (adverse interactions with the police), and structural (poverty-related) stressors and injection drug use and risky injection drug use (e.g.., receptive and non-receptive needle sharing, frontloading and back-loading, or sharing drugs from a common container).

**Results:**

Participants below the poverty line had significantly lower odds of injection drug use (OR 0.52, 95 % CI: 0.27-0.99, *p* = 0.047) and risky injection drug use behavior (OR 0.48, 95 % CI: 0.25-0.93, *p* = 0.030). In addition, participants with an arrest history had higher odds of injection use (OR 19.58, 95 % CI: 9.81-39.10, *p* < 0.001) and risky injection drug use (OR 16.25, 95 % CI: 4.73-55.85, *p* < 0.001). Participants with depression had significantly higher odds of engaging in risky injection drug use behavior (OR 3.26, 95 % 1.39-7.67, *p* = 0.007). Focusing on participants with a history of injection drug use, we found that participants with depression were significantly more likely to engage in risky drug use compared to participants below the depression cutoff (OR 3.45, 95 % CI: 1.23-9.66, *p* < 0.02).

**Conclusions:**

Findings underscore the need to address psychosocial and structural-level stressors among Malaysian fishermen to reduce HIV injection drug risk behaviors.

**Electronic supplementary material:**

The online version of this article (doi:10.1186/s12889-016-3125-7) contains supplementary material, which is available to authorized users.

## Background

It has been estimated that there are 170,000 people who inject drugs (PWID) in Malaysia [1]. HIV prevalence among PWID in Malaysia ranges from 25 % to 45 % [1]. Further, people who inject drugs (PWID) account for 39 % of all new reported cases of people living with HIV in Malaysia, deeming drug risk behavior a serious public health concern in the country [2]. Malaysian fishermen have not only grown by 22 % in the past decade, they have been identified as a key-affected HIV population with rates 10 times higher than national rates [3–5]. Previous HIV research among fishermen has mainly focused on sexual risk behaviors (see review [6]). Recent research examining injection drug use behavior among fishermen is scarce, although growing (e.g.,[7, 8]), and indicates that injection drug use and risky injection drug use (i.e., receptive and non-receptive needle/syringe sharing, frontloading and back-loading, sharing equipment, sharing drugs from a common container, or adding blood to the drug solution before injecting increasing one’s risk of HIV infection) may play a central role in the high rate of HIV among fishermen.

Psychosocial and structural-level stressors may contribute to HIV injection drug risk behavior among this key-affected population. Our study is guided by the ecological framework perspective, which suggests that multi-level factors contribute to an individual’s health and well-being at micro, meso and macro levels [9]. Based on this theoretical approach and empirical research conducted in Western and non-Western settings, depressive symptoms (individual/micro) [10–15], adverse interactions with police (social/meso) [16], and poverty-related stressors (structural/macro) [17–19] have been noted to be associated with HIV injection drug use and injection risk behavior (e.g. rushed injections, needle sharing). Further, while originally developed to understand the pathway between sexual abuse and HIV sexual and drug risk behavior, Miller’s [20] conceptual model may be adapted to understand stressors/potentially traumatic events and HIV drug risk behavior among Malaysian fishermen. The model posits that stressors contribute to the use of maladaptive coping mechanisms leading to HIV risk behaviors [20]. Drug use behaviors may also be understood as a way to self-medicate in response to individual, social, and structural stressors, and may also be more easily available and effective as an immediate coping method [14, 20]. The model also suggests that feelings of hopelessness and despair, which may be associated with stressors at the individual, social or structural levels, are related to unsafe injection practices [14, 20, 21].

Research has identified a number of multi-level stressors, which are associated with injection drug use and risky injection drug use. On an individual level, numerous studies conducted in Western (e.g.,[15, 22, 23]) and non-Western settings (e.g.,[10, 21, 24]) suggest moderate to severe levels of depressive symptoms among injection drug users. Data also suggests that PWIDs are at risk for suicidal ideation and suicide attempts [10, 25, 26]. Research has also indicated a consistent relationship between depressive symptoms and risky injection drug practices [15, 27–29]. For example, in a meta-analysis, Conner and colleagues [27] found a significant relationship between depression and needle sharing among injection drug users, and suggests that moderate to severe levels of depression may increase needle sharing due to feelings of hopelessness or as an attempt to cope with negative emotions by engaging in social interactions with others.

At the social level, the association between police interactions and risky injection practices, such as rushed injections, needle sharing and use of shooting galleries, has been noted among a number of studies [17, 30, 31]. Specifically, risky injection practices and HIV prevalence are associated with arrests by police and police removal of syringes [32–34]. This may be due to rushed and hurried injections without clean needles for fear of coming in contact with the police or an increased likelihood in borrowing needles from others when police remove syringes [35]. A perceived increase in police presence was noted to be a risk factor for midazolam injection among injection drug users in Thailand [36]. Unlawful harassment and abuse by police has also been noted to increase risky injection practice among injection drug users [37, 38]. Similar to depression, abuse by police may increase the likelihood of risky drug injection practices due to feelings of hopelessness and despair as a result of trauma and increase maladaptive coping mechanisms [20]. As such, it is critical to examine arrests and abuse by police and the relationship with risky injection drug use practices among the key-affected population of Malaysian fishermen.

At the structural level, a number of studies have indicated that poverty-related stressors, (i.e., substandard housing conditions, homelessness, and food insecurity) are related to injection drug use [17–19]. Similar to the relationship with symptoms of depression, injection drug use may be a way to cope with daily and ongoing financial stressors. Substandard housing and homelessness has been found to be associated with both injection drug use [39–42] and engaging in risky drug use behaviors such as needle sharing [10, 18, 19]. Further, homeless PWIDs are more at risk for HIV infection compared to those who are housed [19]. In a U.S based longitudinal study among PWIDs, Aidala and colleagues [39] found that a change from homelessness to sufficient housing significantly reduced risk of injection drug use, needle use and needle sharing. Additionally, Weiser and colleagues [43] found a relationship between food insecurity and HIV risk behavior. Poverty-related stressors, namely homelessness, poverty, financial stress and food insecurity have not been examined specifically among Malaysian fishermen. As poverty-related stressors have been noted to be associated with injection drug use and risky drug use practices among other populations, it is critical to understand this relationship among this key HIV-affected population for prevention efforts.

The aim of this paper is to address the gap in research on depression, policing and poverty-related stressors as predictors for injection drug use and risky injection drug use among Malaysian fishermen. Examining stressors at the individual, social, and structural level among Malaysian fishermen will contribute to the development of more effective prevention and intervention efforts, which will potentially address multiple risk factors for HIV. As such, in the current study, we examine: 1) the prevalence of depression, policing, poverty-related stressors (i.e., homelessness, poverty and food insecurity), injection drug use, risky injection drug use, and HIV infection among a sample of 406 Malaysian fishermen; 2) the relationship between depression, policing, poverty and injection drug use and risky injection drug use among this sample of Malaysian fishermen; and 3) the relationship between depression, policing (specifically, adverse interactions with police), poverty-related stressors and risky injection drug use among a sub-sample of 154 Malaysian fishermen with a history of injection drug use. Based on previous research among other populations, we hypothesize that after controlling for demographic variables, depression, policing and poverty will predict injection drug use and risky injection drug use among the sample. Further, among fishermen who endorse a history of injection drug use, we hypothesize that after controlling for demographic variables, depression, experiencing abuse by police and poverty will be associated with HIV risky injection drug use behaviors.

## Methods

This paper used data from Project WAVES, a project designed to assess HIV prevalence, risk behavior, and characteristics of HIV risk behaviors among fishermen in Malaysia. The study utilized a mixed methods approach with in-depth interviews and a quantitative cross-sectional survey collected between 2009 and 2011. The current study uses data solely from the quantitative survey collected from July to December 2011.

### Study population

In 2009, 3,720 men were registered fishermen in Kuantan [3]. However, these numbers do not include those who are unregistered and unlicensed in the region, typically small-scale artisanal fishermen. As there is no universal definition and agreement regarding what is deemed a small-scale fishery, there is consensus in the underestimation of the number of small-scale fishermen [44]. Globally, the prevalence of small-scale artisanal fishermen is understood to be significantly larger than large-scale commercial fishermen and contribute to more than half of fish catch worldwide [45].

### Study site

The study was conducted in Pahang state, which is around the Kuantan jetty*.* This is one of the busiest fishing jetties in the country and central to commercial and small-scale fishing in Malaysia. In 2006, Kuantan was one of the first towns in Malaysia to implement the Needle and Syringe Exchange Program deeming this an appropriate and relevant place for the study. Sampling locations comprised two government-owned commercial fishermen’s wharfs in Kuantan and a fishing village within a 100 KM radius of Kuantan.

### Sampling and data collection procedures

Recruitment for the survey was based on Respondent Driven Sampling (RDS), a network, chain referral sampling methodology utilized for the recruitment of hard to reach, vulnerable or mobile populations [46, 47]. While fishermen, in general, are not a hidden population per se, they are highly mobile. There is also no adequate sampling frame that exists for fishermen in Malaysia that includes both large and small-scale fisheries. Current official lists are restricted to Malaysian vessel owners (both commercial and non-commercial with no database specifically capturing non-vessel owning fishermen (i.e. deckhands aboard vessels) as well as non-Malaysian nationals who tend to operate illegally. Furthermore, for the purpose of this estimation, it is important to adequately recruit fishermen who are involved in illicit (i.e., substance use) behaviours, which may not be easily captured by standard surveillance methods [48].

High mobility and the stigma of illicit behaviors makes sampling a challenge among Malaysian fishermen. Respondent-driven sampling (RDS), a coupon-based chain-referral method, was used in the current study to address some of the aforementioned obstacles [46, 48–50]. RDS was chosen as an economical and time-efficient recruitment method which has been successful in recruiting PWID [51–53]. In addition, RDS uses a statistical technique that adjusts for potential over-reliance on characteristics if the initial sample and bias towards participants who may be typically cooperative [49]. Informed by data from the social network of the participants, prevalence estimates are determined of specific traits in the target population [50].

In RDS, a small number of participants are recruited as ‘seeds,’ representing the characteristics of the population of interest and socially well-connected to the target population. After the seeds complete the survey, they are then provided a fixed number of coupons (3 in our study) to distribute to social network members who meet inclusion criteria. The coupon is required for screening into the study after the initial seed. Each participant is then subsequently provided the same number of coupons. Data are collected in the social networks of each participant with anonymous identification numbers to link the recruitment chains throughout the study. With each wave, new recruits become more independent of the index participants of each wave, decreasing bias and eventually reaching equilibrium [46, 49].

In the current study, eight seeds were recruited based on their motivation to participate and how socially connected they were to the fishing community. Three initial seeds reported they were fishermen who used drugs and three reported non-drug use. Two additional drug-using fishermen seeds were added because of lack of recruitment by two of the initial seeds. Recruited participants who completed the survey received RM50 as compensation for their time. Upon completion of the survey each participant received three coupons to recruit other fishermen. As secondary incentive, the recruiting participant received an additional RM25 for a successful recruit.

Eligibility criteria included being: a) male, b) 18 years or older, c) working as a fisherman full-time (six months or more in the past year), d) speaks Malay, and e) provided written informed consent. At each sampling location, a person who injected drugs (PWID) and a non-injector were recruited (with a total of six initial participants). Every participant who completed the study received three coupons to recruit peers from their social network. Recruitment was complete when the sample reached equilibrium, which was determined when key variables (HIV, injection drug use status, and time spent at sea) changed by less than 2 % between recruitment waves [46]. This was determined from previous research which indicates that the leading wave approximates equilibrium within 2 % after 6 waves when respondents recruit three peers [46, 49].

As previously noted, quantitative data for Project Waves were collected between July and December 2011. The questionnaire was self-administered on a laptop computer using Questionnaire Development System (QDS) software (Nova Research Company, Maryland, USA). All questions were in Malay. All data for the current study can be found in the Additional File 1.

### Measures

All sections of the survey were translated to Malay and pilot tested among fishermen from Kuantan, Pahang to ensure both face and content validity of the measures.

### Demographic variables

Demographic variables examined in this study included, age, ethnicity, marital status, religion, and education. Participants were also characterized by the type of vessel in which they worked (commercial vs. traditional) and their occupational role (captain vs. deckhand). Working on a commercial vessel indicated large-scale fisheries. All demographic variables were dichotomized.

### Depression

Depression symptoms were assessed using the Brief Symptoms Inventory (BSI; [54]) 6-item depression subscale (including symptoms related to dysphoric mood and affect, lack of motivation, and loss of interest in life). Participants were asked how much each symptom bothered them in the past 7 days on a Likert scale from 0 = not at all to 4 = extremely. Scores from the 6 items were summed into a total score and then converted into a T-score with a mean of 50 and standard deviation of 10. A cutoff of 63 or above indicated severe depressive symptoms, based on previous research with a normative non-patient population [55].

The BSI depression subscale has been used widely among adults in both western [56, 57] and non-western settings [56, 58]. Previous research has demonstrated adequate internal reliability of the depression subscale ranging from Cronbach’s alpha = 0.85 [54] to 0.96 [59]. The BSI has also demonstrated adequate criterion and construct validity [54]. In the current study, reliability was good with Cronbach’s alpha = .85 (one-sided 95 % confidence interval = .83).

### HIV

During the informed consent process, individuals were informed that the study would involve a rapid test for HIV. Willingness to undergo HIV testing was an eligibility criterion for enrolling in the study. All participants underwent pre-test and post-test counselling by research assistants trained in HIV counselling, testing, and referral. Participants were also asked if they had been previously tested for HIV and, if so, asked the result of their most recent test. HIV serology was determined using a rapid test (ACON HIV Rapid Test kit, ACON Laboratories, California, USA); reactive results were confirmed with a second rapid test (Intec Products Inc., Xiamen, China). Two reactive results were required for subjects to be classified HIV positive. Two participants had discordant results between the two tests and were classified as HIV negative.

### Policing

Participants were asked if they had “*ever been arrested”* (with 0 = never been arrested and 1 = been arrested 1 or more times). Among those who reported a prior arrest, the “*number of times arrested”* (continuous variable), as well as whether they had “*ever been brought to police lock-up”* were asked (with 0 = never been brought to police lock-up and 1 = been brought to police lock-up 1 or more times). Among those who had been brought to police lock up, “*number of days detained”* (continuous variable) was obtained. Participants were also asked if they had “*ever gone to prison*” (with response categories of 0 = never gone to prison and 1 = been to prison 1 or more times) and, if so, the “*number of times been to prison*.”

Participants who endorsed “*any previous drug use”* were also asked an additional 7 questions related to negative police interactions. These additional questions were formulated for the Project Waves study and based on formative qualitative research we conducted among fishermen in the Kuantan region. Specifically, participants were asked if “*fear of the police ever caused a hurried or rushed injection”* (0 = No and 1 = Yes), if “*the police ever demanded money to buy back drugs that had been confiscated”* (0 = No and 1 = Yes), if they had “*ever been beaten or tortured by the police”* (0 = No and 1 = Yes), if the “*police ever planted drugs on them”* (0 = No and 1 = Yes), if the “*police ever took their syringes”* (0 = No and 1 = Yes), if the participant ever “*avoided carrying syringes for fear of the police”* (0 = No and 1 = Yes), and “*in the past 6 months, the number of times the boat or place of work was raided by the police”* (0 = No and 1 = Yes).

### Poverty-related stressors

Poverty-related stressors included: “*being at or below the poverty line”* (i.e., a monthly income at or below RM820, approximately 273 USD), or “*borrowing money in the past 3 months.”* In addition, food insecurity was defined as *“having enough money to buy food in the past 3 months.”* Homelessness was defined as “*having a consistent place to stay in the past 3 months*.” All poverty-related variables were dichotomized (No = 0, Yes = 1).

### Injection drug use

Injection drug use in our study was defined as ever intravenously injecting a substance. Injection drug use was determined using a series of questions covering 8 distinct substances, with an additional “other” category. Specifically, participants were asked if they had ever injected the following: 1) Subutex, Suboxone, Buprenorphine; 2) Ketamine; 3) Pil kuda; 4) heroine; 5) Ice, Syabu Crystal Meth; 6) Methadon; 7) Ecstasy; and 8) Dormicum, Benzodiazapene. Each respondent was asked whether they had ever injected the drug, and was coded as ‘1’ if they reported injecting ANY drug (PWID) and ‘0’ if they had never injected any of the substances (non-injector).

### Risky injection drug use

Risky injection drug use behaviors were assessed among participants who endorsed injection drug use. Risky injection drug use behavior was defined as unsafe practices when intravenously injecting a substance. Unsafe injection practices in the past month was a dichotomous measure based on the Risk Behaviour Assessment [60], which asks a series of eight questions on injection-related risk, covering receptive and non-receptive needle/syringe sharing, frontloading and back-loading, sharing equipment, sharing drugs from a common container, or adding blood to the drug solution before injecting. The respondent was coded as ‘1’ if they had engaged in any of these behaviors one or more times in the past month, indicating unsafe injection practices, and ‘0’ if they reported zero times for all behaviors.

### Data analysis

All analyses were conducted in STATA SE13 [61]. Fisher’s exact tests or χ^2^ tests were used to examine group differences among those who reported injection drug use and those who reported no injection drug use for categorical variables, and t-tests were used to examine differences between groups for continuous variables. Missing data was minimal across all measures; the vast majority had 0 % missingness, with a few variables that had 1 % missingness due to nonresponse. Where missing, figures are reported using available data. All variables of interest were examined as potential confounders (e.g., HIV status and depression) and all predictors were assessed for multi-colinearity before being entered in multivariate logistic regression models. Any variables that were collinear were removed and one variable was chosen as a marker for the construct. Multivariate logistic regressions were conducted controlling for demographic variables with psychosocial and structural-level HIV risk factors as predictors of injection drug use behavior and risky injection drug use (compared to both non-risky injection drug users and non-injection drug users). Finally, a sub-sample multivariate logistic regression was conducted among injection drug users examining psychosocial and structural-level HIV risk factors as predictors of risky injection drug use behavior (compared to non-risky injection drug use behavior only). Individual sampling weights based on self-reported network data were not used for the analyses of the current study because regression is fairly immune to weights [62].

## Results

### Socio-demographic characteristics

A total of 406 fishermen (including 8 seeds) were recruited, with the longest chain in the sample having over 15 recruitment waves, well in advance of the 6 waves needed to achieve equilibrium. The sample was composed of 8 isolated recruitment chains, including two large chains that made up almost 64 % of the data.

Table 1 presents participant socio-demographic characteristics of the total sample (*N* = 406) and bivariate analyses comparing non-injectors (*n* = 252) with PWID (*n* = 154). The majority of the sample was older than 25 years old (85.2 %), Malay (98.8 %), single (63.5 %) and Muslim (98.8 %). Additionally, the majority of the participants reported having completed some secondary education or less (68.7 %), and about 85 % of the study sample reported being a deckhand. Finally, more than half (58.1 %) of the participants reported working on a traditional vessel. Bivariate analyses of socio-demographic variables by injection drug use behavior indicated that being older than 25, single, in a deckhand role, and on a commercial vessel type were significantly associated with injection drug use (all *p* < 0.01).

**Table 1:** Socio-demographics of total sample and by injection drug use behavior

	*Total sample*		*Non-Injector*		*PWID*	
	*(n = 406)*		*(n = 252) (62.1 %)*		*(n = 154) (37.9 %)*	
	n	%	n	%	n	%
Age ^a^, 25 and younger (ref: older than 25)	60/405	14.8 %	54/251	21.5 %**	6	3.9 %
Ethnicity, Malay (ref: non-Malay)	401	98.8 %	250	99.2 %	151	98.1 %
Marital Status, Married (ref: single)	148	36.5 %	109	43.3 %**	39	25.3 %
Religion, Muslim (ref: non-Muslim)	401	98.8 %	250	99.2 %	151	98.1 %
Education, Some secondary or lower (ref: completed secondary or higher)	279	68.7 %	169	67.1 %	110	71.4 %
Employment role/job ^a^, Deckhand (ref: Captain)	341/402	84.8 %	194/248	78.2 %	147	95.5 %**
Vessel type a, Traditional (ref: Commercial)	234/403	58.1 %	185/249	74.3 %**	49	31.8 %

^a^total sample = 406; missingness due to non-response

** *p* <0.01; *p* <0.05; *p* < 0.1

### Individual, social and structural stressors

Table 2 presents individual, social and structural stressors for the sample, and bivariate analyses comparing PWID to non-injectors. Analyses indicated that, nearly 12 % of the study sample tested positive for HIV and that about 10 % of participants scored at or above the cutoff for depression. Poverty-related variables indicated that a majority of participants were at or below the poverty line (71.6 %) and did not have enough money to buy food in the past 3 months (71.2 %). Conversely, the majority of participants reported not having recently borrowed money (75.9 %) and having had a consistent place to stay in the past 3 months (86.5 %). In examining police interactions, a little over half of participants reported having been arrested (50.7 %) with 93.2 % of them reporting having been subsequently brought to police lock-up. Sixty-four percent of the study sample also reported having been to prison, with a mean number of 3.2 (SD = 2.8) times.

**Table 2:** Risk factors among total sample and by injection drug use behavior

	*Total sample*		*Non-Injector*		*PWID*	
	*(n = 406)*		*(n = 252) (62.1 %)*		*(n = 154) (37.9 %)*	
	n	%	n	%	n	%
HIV+	48	11.8 %	8	3.2 %	40	26.0 %**
Mental Health						
-Depression (BSI, cutoff score 63)	42	10.3 %	16	6.3 %	26	16.9 %**
Financial Stressors ^a^						
-At or below poverty line (ref: above poverty line)	288/402	71.6 %	200/248	80.6 %**	88	57.1 %
-Borrowed money in past 3 months (ref: not borrowed)	98	24.1 %	48	19.0 %	50	32.5 %**
-Enough money to buy food in past 3 months (ref: not enough)	117	28.8 %	66	26.2 %	51	33.1 %
Living conditions						
-Needed or wanted place to stay past 3 months, but did not (ref: had consistent place to stay)	55	13.5 %	31	12.3 %	24	15.6 %
Negative Police Interactions						
Ever been arrested	206	50.7 %	66	26.2 %	140	90.9 %**
# times been arrested among those who reported prior arrest (*n* = 206) (mean, sd)	4.3, 4.7		3.3, 3.8		4.8, 5.0*	
Ever been brought to police lock-up, among those that had been arrested (*n* = 206)	192	93.2 %	53	80.3 %	139	99.3 %**
# Days detained in lock-up, past 3 months (mean, sd) among those detained) (*n* = 190) ^a^	4.6, 6.6		3.3, 5.8		5.1, 6.8†	
Ever gone to prison ^a^	130/203	64.0 %	23/63	36.5 %	107/140	76.4 %**
# times gone to prison (mean, sd) among those previously gone to prison (*n* = 203) ^a^	3.2, 2.8		2.7, 2.3		3.3, 2.9	

^a^missingness due to non-response

** *p* <0.01; * *p* <0.05; † *p* < 0.1

Bivariate analyses indicated that positive HIV status and depression score of 63 or above were significantly associated with injection drug use (both *p* < 0.01). The specific policing variables of arrest history, police lock-up, and prison history were associated with PWID, as well as number of times arrested (all *p* < 0.01), with PWID experiencing a significantly higher number of arrests (M = 4.8, SD = 5.0) compared to non-injector (M = 3.3, SD = 3.8). Interestingly, being at or below the poverty line was significantly associated with being a non-injector (*p* < 0.01), while borrowing money in the past 3 months was associated with PWID (both *p* < 0.01).

### Multivariate logistic regression analyses

A multivariate logistic regression analysis with PWID as the outcome indicated that participants below the poverty line had significantly lower odds of injection drug use compared to those who were above the poverty line (OR 0.52, 95 % CI: 0.27-0.99, *p* = 0.047), in contrast to our hypothesis. In support of our hypothesis, participants who had ever been arrested had higher odds of injection use compared to those who had never been arrested (OR 19.58, 95 % CI: 9.81-39.10, *p* < 0.001). Policing variables were highly correlated, so arrest history was the only policing variable entered into the model. In contrast to our hypothesis, depression, borrowing money, having enough money to buy food, and having a consistent place to stay were not significantly related to injection drug use (all *p* > 0.05), (see Table 3).

**Table 3:** Regression analysis of predictors of injection drug use behavior and risky injection drug use behavior

	Injection drug use^a^			Risky injection drug use^b^		
	*OR*	*95 % CI*	*p value*	*OR*	*95 % CI*	*p value*
Age, 25 and younger (ref: older than 25)	0.13**	0.05–0.38	0.000	0.45	0.12–1.71	0.243
Ethnicity, Malay (ref: non-Malay)	1.74	0.18–17.05	0.633	2.56	0.23–28.37	0.442
Marital Status, Married (ref: single)	0.54†	0.28–1.05	0.068	0.7	0.33–1.47	0.346
Education, Some secondary or lower (ref: completed secondary or higher)	0.68	0.35–1.35	0.275	0.67	0.33–1.37	0.275
Employment role/job, Deckhand (ref: Captain)	2.72†	0.94–7.93	0.066	0.77	0.24–2.46	0.660
Vessel type a, Traditional (ref: Commercial)	0.28**	0.15–0.51	0.000	0.5*	0.25–0.99	0.048
Depression (BSI, cutoff score 63)	1.92	0.69–5.36	0.214	3.26**	1.39–7.67	0.007
At or below poverty line (ref: above poverty line)	0.52*	0.27–0.99	0.047	0.48*	0.25–0.93	0.030
Borrowed money in past 3 months (ref: not borrowed)	1.15	0.57–2.30	0.702	1.94†	0.97–3.88	0.062
Enough money to buy food in past 3 months (ref: not enough)	1.05	0.54–2.03	0.890	1.26	0.63–2.53	0.517
Needed or wanted place to stay past 3 months, but did not (ref: had consistent place to stay)	1.38	0.59–3.24	0.547	1.69	0.71–4.02	0.234
Ever been arrested	19.58**	9.81–39.10	0.000	16.25**	4.73–55.85	0.000

** *p <* .01; * *p <* .05; † *p* < 0.1

^a^ reference group: non-injectors

^b^ reference group: all others (i.e. non-risky injection drug users and non-injectors)

Findings from the multivariate logistic regression analysis comparing risky injection drug users (*n* = 66) with combined non-risky injection drug users and non-injection drug users (*n* = 340) as an outcome yielded similar results. In contrast to our hypothesis, participants at or below the poverty line had significantly lower odds of engaging in risky injection drug use behavior (OR 0.48, 95 % CI: 0.25-0.93, *p* = 0.030) compared to those above the poverty line. In support of our hypothesis, participants who had ever been arrested had significantly higher odds of engaging in risky injection drug use behavior (OR 16.25, 95 % CI: 4.73-55.85, *p* < 0.001). In contrasts to findings with injection drug use as the outcome, in this model, participants with depression at or above the cutoff had significantly higher odds of engaging in risky injection drug use behavior (OR 3.26, 95 % 1.39-7.67, *p* = 0.007). In addition, participants who borrowed money in the past 3 months were more likely to engage in risky injection drug use behavior, although trending on significance (OR 1.94, 95 % CI: 0.97-3.88, *p* < 0.06). Having enough money to buy food and having a consistent place to stay were not significantly related to risky injection drug use behavior (all *p* > 0.05) (see Table 3).

### Psychosocial and structural stressors among PWID

Bivariate analyses among PWID (*n* = 154) examining risky injection drug use compared to non-risky injection drug use indicated that *fearing the police causing a rushed or hurried injection* (74.2 %) was significantly associated with risky injection drug use behavior (*p* = 0.019). With marginal significance, reporting the *police ever taking your syringes* (60.6 %) was significantly associated with engaging in risky injection drug use behavior (*p* = 0.052) (Table 4).

**Table 4:** Policing interactions by risky injection drug use

	*Total sample*		*Non Risky Injection Drug Use*		*Risky Injection Drug Use*	
	*(n = 154)*		*(n = 88) (57.1 %)*		*(n = 66) (42.9 %)*	
	n	%	n	%	n	%
Has fear of police ever caused a hurried or rushed injection	98	63.6 %	49	55.7 %	49	74.2 %*
Have the police ever demanded money to buy back drugs that have been confiscated	46	29.9 %	23	26.1 %	23	34.8 %
Have you ever been beaten or tortured by police	74	48.1 %	37	42.0 %	37	56.1 %
Have the police ever planted drugs on you	43	27.9 %	24	27.3 %	19	28.8 %
Have police ever taken your syringes	79	51.3 %	39	44.3 %	40	60.6 %†
Have you ever avoided carrying syringes for fear of the police	107	69.5 %	58	65.9 %	49	74.2 %
In the past 6 months, how many times has your boat or the place you were working been raided by the police? (mean, sd)	3.67, 10.7		2.4, 4.5		5.3, 15.5†	

^a^missingness due to non-response

** *p* <0.01; * *p* <0.05; † *p* < 0.1

In support of our hypothesis, findings from the logistic regression examining risky injection drug use as the outcome indicated that participants who met the cutoff for depression were significantly more likely to engage in risky injection drug use behaviors compared to participants below the depression cutoff (OR 3.45, 95 % CI: 1.23-9.66, *p* = 0.018). Similar to the other multivariate analyses, participants at or below the poverty line were less likely to engage in risky injection drug use behaviors, although this was marginally significant (OR 0.50, 95 % CI: 0.23-1.07, *p* = 0.075) (Table 5).

**Table 5:** Regression analysis of risky injection drug use behavior compared to non-risky injection drug use

	Risky IDU as outcome		
	*OR*	*95 % CI*	*p value*
Ever tortured by police	1.27	0.60–2.67	0.531
Age, 25 and younger (ref: older than 25)	1.80	0.30–10.89	0.521
Ethnicity, Malay (ref: non-Malay)	4.23	0.26–67.56	0.308
Marital Status, Married (ref: single)	1.03	0.44–2.41	0.951
Education, Some secondary or lower (ref: completed secondary or higher)	0.69	0.31–1.55	0.371
Employment role/job, Deckhand (ref: Captain)	0.20†	0.3–1.34	0.097
Vessel type a, Traditional (ref: Commercial)	1.06	0.47–2.38	0.897
Depression (BSI, cutoff score 63)	3.45*	1.23–9.66	0.018
At or below poverty line (ref: above poverty line)	0.50†	0.23–1.07	0.075
Borrowed money in past 3 months (ref: not borrowed)	1.99†	0.89–4.47	0.095
Enough money to buy food in past 3 months (ref: not enough)	1.2	0.52–2.78	0.667
Needed or wanted place to stay past 3 months, but did not (ref: had consistent place to stay)	1.54	0.57–4.15	0.398
Ever been arrested	2.71	0.66–11.15	0.167

** *p <* .01; * *p <* .05; † *p* < 0.1

## Discussion

This study examines psychosocial stressors and injection drug use among Malaysian fishermen. Results show that Malaysian fishermen experience a range of ongoing and deleterious psychosocial and structural stressors. Most of the fishermen in the current study reported living at or below the poverty line and did not have enough money to buy food. This is consistent with other studies among fishermen in LMICs, showing fishermen at high risk for extreme levels of poverty [5]. Furthermore, about 10 % of the study population met the criteria for depression, almost double that of the12-month prevalence (5.6 %) found in other low and middle income countries, based on data collected from a cross-national survey in 18 countries [63]. Additionally, over half of the fishermen reported prior arrests and subsequent imprisonment on multiple occasions. Among injection drug users, we found that the majority feared the police, causing hurried or rushed injections and avoidance behaviors in carrying syringes, which exacerbates HIV risk. Alarmingly, we also found that almost half of the injection drugs users in our sample had been beaten or tortured by the police. Taken together, these psychosocial stressors of poverty, depression, and policing suggest a critical need to address these needs and develop appropriate services for fishermen in Malaysia.

### Depression and injection drug use

Depression was not associated with injection drug use, inconsistent with previous research in both Western (e.g., [15, 22, 23]) and non-Western settings (e.g.,[10, 21, 24]). However, depression among Malaysian fishermen was significantly related to risky injection drug use behavior. Further, depression was the main driver of risky injection drug use among injection drug users in our analyses specifically among PWID. These findings are consistent with research, which has noted a relationship between depressive symptoms and risky injection drug practices [15, 27–29, 64]. Results suggesting depression is related to risky injection drug use behavior have serious implications for the overall health and well-being of fishermen. Injection drug users at risk for depression and suicide [10, 25, 26] can be extremely dangerous in environments where the means to fatally harm oneself is readily available (namely through drugs). Further, risky injection drug use increases the risk for HIV infection among fishermen, a population already severely impacted by the HIV epidemic [5, 65].

### Policing and abuse by police and injection drug use

History of arrest is associated with both injection drug use and risky injection drug use behavior. This is in line with previous research, which has demonstrated the association between risky injection practices with arrests by police and police removal of syringes [32, 33]. This is an important finding as HIV prevalence among injection drug users has been found to be associated with arrests and police presence [32, 34]. Further, research has indicated that police arrests are associated with an increased risk of non-fatal drug overdoses [66] and a decreased use of syringe exchange programs [67] among injection drug users.

Results from our multivariate sub-analyses did not find experiencing torture or physical violence by the police as a significant predictor of risky injection drug use behavior. This is inconsistent with previous research which has found unlawful harassment and abuse by police to increase risky injection practice among injection drug users [37, 38]. Similar to depression and in line with the conceptual model of Miller [20], trauma experienced by police may increase the likelihood of risky drug injection practices through the use of maladaptive coping mechanisms. Perhaps, in our study, symptoms of depression were a result of abuse by the police, which was the driving predictor of risky injection drug use behavior. Future research should examine specific trauma outcomes as a mediator of abuse by the police in order to understand the nature of this relationship. Harsh policies regarding drug use in Malaysia including flogging and imprisonment for drug possession, imposed rehabilitation and forced testing for drug use [68], may increase police presence and harassment among Malaysian fishermen. As such, it is critical to examine the potential outcomes of adverse interactions with police and the relationship with risky injection drug use practices among this key-affected population.

### Poverty-related stressors and injection drug use

In the current study, poverty-related stressors were inconsistently related to injection drug use and risky injection drug use behavior. Specifically, food insecurity and homelessness did not increase the likelihood of injection drug use or risky drug use behavior. This is in contrast to previous literature, which has demonstrated injection drug use as a coping mechanism in dealing with homelessness [39, 40, 42] and food insecurity [43]. However, there may be factors specifically related to the cultural context of being a fisherman in Malaysia which contributes to the lack of association in our study. For example, frequent mobility resulting in long periods at sea, as well as access to staying on the boat, may reduce stress associated with housing needs and subsequent injection drug use. In addition, being a part of a wide network of other fishermen may increase access to food and other resources even if one does not have enough money to buy their own, thereby reducing stress and subsequent injection drug use.

Results from our study indicate only marginal significance of risky injection drug use among fishermen who borrowed money in the past 3 months compared to those who did not. Perhaps fishermen in our study felt more stress and helplessness in borrowing money, increasing the likelihood of risky injection drug use behaviors. Borrowing money may be a more formal interaction among fishermen thereby increasing stress, whereas sleeping at a colleague’s residence or sharing food is seen as less formal. Further, fishermen who ask to borrow money may be more likely to borrow money specifically to purchase injection drugs and may be in a social group where injection drug use and borrowing money is common and acceptable. Previous analyses from this study found that risky injection drug use behavior was associated with having a large proportion of drug injectors in one’s network [8] providing some evidence to support this conclusion.

Also in contrast to our hypothesis, fishermen at or above the poverty line were more likely to engage in injection drug use, risky injection drug use, as well as risky injection drug use among the sub-sample of only injection drug users. Over 70 % of the study sample was below the poverty line, suggesting a sample with very limited access to financial resources. Perhaps only those with relatively more financial access are in the position to buy injection drugs. Also, being on a commercial boat predicted both injection and risky injection drug use behavior. Fishermen on commercial boats tend to be at sea longer and make more consistent income. Findings from previous analyses suggest that injection drug use was prevalent on the boat, often with the captain and other crewmembers aware of the occurrence [8]. In addition, having a captain provide drugs for work on the boat was common [8]. This suggests that this context provides fishermen with the means to regularly engage in injection drug use. Not surprisingly, deckhands were marginally more likely to engage in injection drug use, but not risky injection drug use. This could be due to the fact that captains who provide drugs for their crewmembers are more likely to provide access to clean needles/syringes [8]. This suggests that both consistent financial resources, others around you who inject drugs, and longer time at sea may contribute to our understanding of financial stressors in the relationship to injection drug use. As this is the first study we know to examine financial stressors related to injection drug use among fishermen in Malaysia, future research should continue to build upon our understanding taking into account contextual factors which may contribute to this relationship.

Findings of the current study indicate extremely high rates of financial difficulties, symptoms of depression, and adverse interactions with the police, suggesting an environment with a heavy burden of stress. Overall, our findings highlight a complex relationship between psychosocial and structural stressors and both injection drug use and risky injection drug use behaviors among Malaysian fishermen, underscoring the need for more research in this area. Our results demonstrate depression is a major driving factor in predicting risky injection drug use behavior among Malaysian fishermen. Other mental health symptoms, such as anxiety, post-traumatic stress and local idioms of distress should be examined among this population as mental health problems have been found to be associated with injection drug use and risky injection drug use behaviors among other populations [10, 24]. Future research should also examine the relationship between psychosocial stressors, injection drug use, and other structural factors such as network size, mobility, social support, and access to services to further delineate the interplay between micro-, mezzo-. and macro-level factors among fishermen in Malaysia.

### Strengths and limitations

This is the first study we know of to examine individual, social, and structural level stressors in association with injection drug use and risky injection drug use behaviors among Malaysian fishermen. As a growing key-affected population at risk for HIV infection, we believe our findings are an important contribution to the field and can potentially inform the development of relevant HIV intervention prevention efforts among Malaysian fishermen. However, there are limitations to the study which should be noted. With the exception of HIV testing, we relied on self-report for the study, which may have introduced bias in assessing risky personal and potentially illegal behaviors. Also, as a cross-sectional study, the causal direction between interactions with the police and injection drug use/risky injection drug use is unknown. It may be that fishermen in Malaysia who inject drugs or engage in risky injection drug use behaviors are more likely to be arrested. Similarly, symptoms of depression may be an outcome of risky injection drug use behavior rather than the cause. Future research should examine the causal relationship utilizing longitudinal methods in order to disentangle the nature of this relationship. In addition, as Kuantan was one of the first towns in Malaysia to implement the Needle and Syringe Exchange Program, the potential impact of the program on study participants may have influenced the results. Finally, the current study used respondent driven sampling methods in order to access and recruit a highly mobile and at risk population involved in illicit behaviors. However, reliance on self-report of network size through RDS can potentially produce bias in the estimates [69, 70], limiting our ability to generalize our findings to larger fishermen population of Malaysia.

## Conclusions: implications for HIV prevention

Given the high rates of negative interactions with police, depression, and financial stressors among the fishermen in this sample, our study demonstrates the critical need for the development of HIV intervention prevention efforts which address these psychosocial and structural level stressors among Malaysian fishermen who inject drugs. HIV prevention efforts among Malaysian fishermen should especially be targeted among those who engage in risky injection drug use. Integrating screening and referrals for depression in the current implementation of needle exchange programs should be considered to reduce HIV injection drug risk behavior and symptoms of depression. Further, the development of an onsite center at the ports for needle exchange and depression screening for fishermen should be explored. Addressing mobility constraints for HIV prevention through mobile technology has been deemed complicated among other mobile populations [71], but should be explored as a potential effective tool to reduce HIV risk behavior. Our study also indicates the need to develop structural-level changes in policing, which were found to contribute to risky injection drug use among Malaysian fishermen. Novel alternative policing practices and interventions, such as referrals to rehabilitation programs, rather than arrests may decrease risky injection drug use among Malaysian fishermen. Our findings suggest that HIV prevention efforts should target Malaysian fishermen with financial resources and longer trips at sea, perhaps through saving-programs, which may reduce access to injection drugs and risky injection drug use among this population. As research has indicated that Malaysian fishermen engage in injection drug use behavior with other fishermen at sea [8], potential HIV preventions which are peer led should be explored. Overall, our findings suggest the need for the development of HIV prevention interventions to address multi-level stressors which contribute to HIV injection drug use and HIV risky injection drug use behavior among this vulnerable and key-affected population.

## Abbreviations

BSI, brief symptom inventory; IDU, injection drug use; LMIC, low and middle income country; OR, odds ratio; PWID, people who inject drugs

## Additional file

Additional file 1: Waves_dataset. (DTA 4047 kb)
